# Case Report: Laparoscopic-endoscopic cooperative full-thickness resection for recurrent transverse colon cancer after rectal cancer surgery: a case report with video

**DOI:** 10.3389/fonc.2026.1908320

**Published:** 2026-08-07

**Authors:** Zikai Zhou, Qingbin Wu, Wenjian Meng

**Affiliations:** 1 Colorectal Cancer Center, Department of General Surgery, West China Hospital, Sichuan University, Chengdu, China; 2 West China School of Medicine, Sichuan University, Chengdu, China

**Keywords:** case report, colon cancer, function-preserving surgery, laparoscopic and endoscopic cooperative surgery, recurrent colorectal lesion

## Abstract

A 61-year-old man with a history of rectal cancer surgery developed a recurrent lesion in the transverse colon after repeated endoscopic resections. Histopathology initially showed high-grade intraepithelial neoplasia and subsequently intramucosal adenocarcinoma with negative deep margins, yet the lesion recurred within 1 month. Histopathological initially showed high-grade intraepithelial neoplasia and subsequently later intramucosal adenocarcinoma with negative deep margins, yet the lesion recurred within 1 month. At our institution, colonoscopy demonstrated a 2.0 cm elevated lesion, whereas cross-sectional imaging revealed neither regional lymphadenopathy nor distant metastatic disease. Because the rapid recurrence was inconsistent with the apparently favorable pathological findings, laparoscopic-endoscopic cooperative full-thickness resection was undertaken to achieve definitive excision and pathological assessment while maintaining bowel continuity. Examination of the resected specimen disclosed moderately differentiated adenocarcinoma extending into the subserosa (pT3), with negative margins, low-grade tumor budding, no lymphovascular or perineural invasion, and retained mismatch repair protein expression. Although oncologic colectomy with regional lymphadenectomy remains the standard treatment for pT3 colon cancer, completion colectomy was omitted after multidisciplinary review and shared decision-making. This exceptional decision reflected the absence of radiographic nodal or distant disease, favorable pathological features, the patient’s previous ultra-low colorectal anastomosis, the anticipated functional burden of further surgery, and his informed preference. At approximately 21 months of follow-up, no recurrence had been detected. However, occult nodal disease cannot be excluded, and this approach should not be considered an alternative to standard oncologic surgery.

## Introduction

Endoscopic resection provides durable local control for most colorectal neoplasms; nevertheless, a minority of lesions recur despite apparently complete excision (1, 2). Rapid regrowth that is disproportionate to apparently favorable histopathological findings may indicate that a deeper invasive component was not captured in the original specimen.

When deep mural invasion or a clinically meaningful risk of nodal metastasis is suspected, oncologic colectomy with regional lymphadenectomy is the standard treatment (3, 4). Laparoscopic-endoscopic cooperative full-thickness resection (LECS) may provide en bloc full-thickness excision for selected complex colorectal lesions while avoiding unnecessary bowel resection (5). However, unexpected pT3 disease after local full-thickness resection creates an oncological dilemma because regional lymph-node staging has not been performed (6, 7). Any decision to omit completion colectomy must therefore be regarded as an exceptional, individualized MDT decision rather than an alternative to standard oncologic treatment.

We report a transverse-colon lesion identified synchronously with rectal cancer that recurred rapidly after repeated endoscopic resections following rectal cancer surgery. The distinctive feature of this case is the sequence of superficial pathological understaging, unexpected pT3 adenocarcinoma in the definitive specimen, and subsequent multidisciplinary deliberation regarding completion colectomy in a patient with a previous ultra-low colorectal anastomosis. This case illustrates the potential diagnostic significance of rapid recurrence and the complexity of management when definitive pathology is substantially more advanced than anticipated.

## Case description

**Figure 1:**
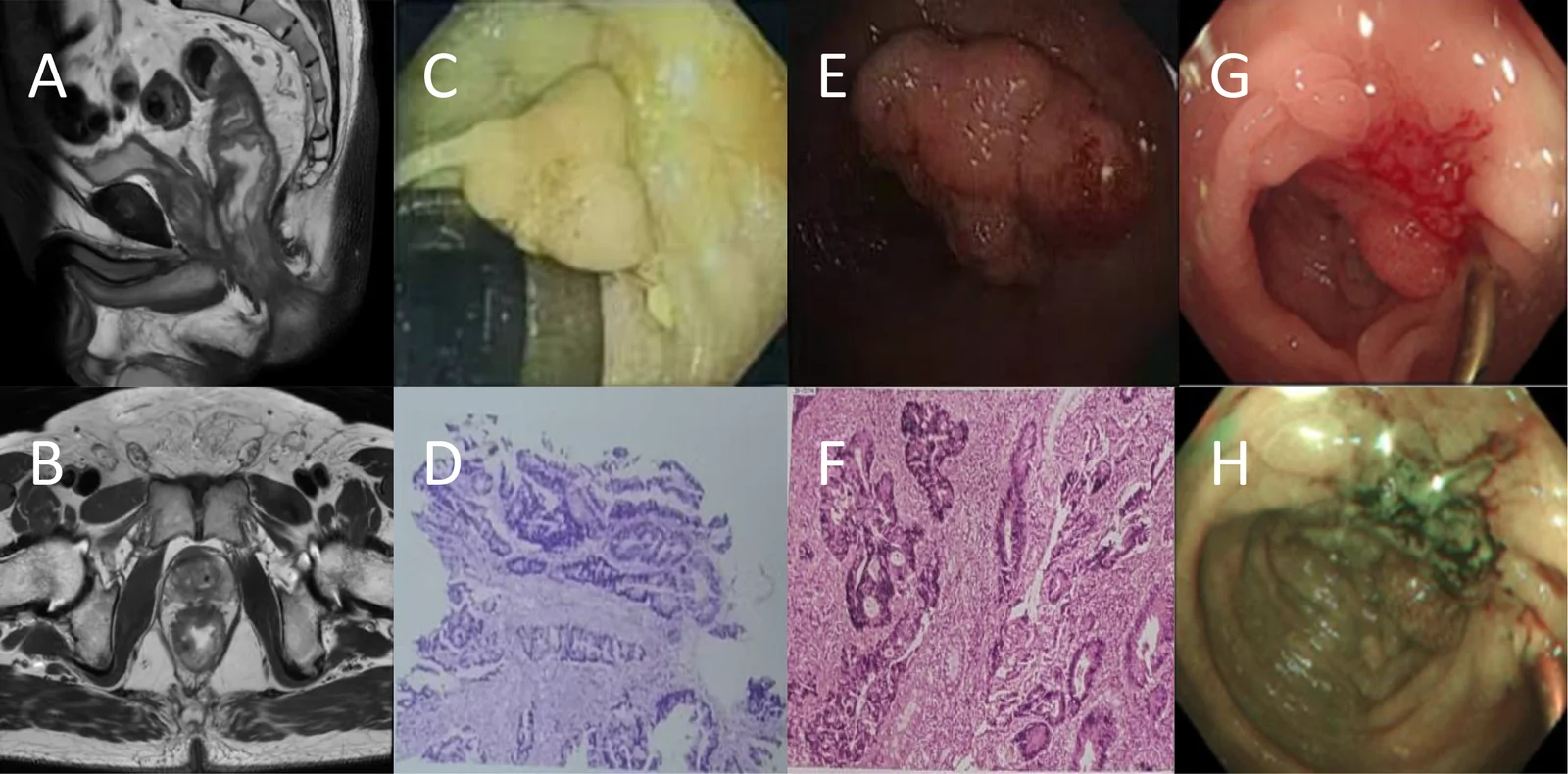
**(A, B)** Preoperative pelvic magnetic resonance imaging (MRI) demonstrating a ycT3aN0M0 rectal lesion (April 2024). **(C)** Initial colonoscopic appearance of the transverse colon lesion (November 2023). **(D)** Histopathological findings from the initial endoscopic biopsy, demonstrating high-grade intraepithelial neoplasia (November 2023). **(E)** Colonoscopic appearance of the lesion at follow-up examination (August 2024). **(F)** Histopathological findings from the endoscopic resection specimen, revealing a tubular adenoma with focal high-grade intraepithelial neoplasia and intramucosal well-differentiated tubular adenocarcinoma (August 2024). **(G)** Colonoscopic appearance of the recurrent transverse colon lesion at repeat examination (October 2024). **(H)** Narrow band imaging of the transverse colon lesion at the repeat colonoscopy (October 2024).

A 61-year-old man presented with cT3aN1M0 low rectal adenocarcinoma and a synchronous transverse colon lesion that had been diagnosed on biopsy as high-grade intraepithelial neoplasia. Following four cycles of neoadjuvant XELOX, he underwent laparoscopic low anterior resection with ileostomy in April 2024. A colorectal anastomosis was constructed approximately 2 cm proximal to the anal verge. Pathological examination demonstrated tumor downstaging to ypT2N0M0 (**Figures 1A, B** and **Supplementary Video 1**), after which two additional cycles of XELOX were administered.

Surveillance of the transverse colon lesion continued at an outside institution. Endoscopic resection performed in August 2024 yielded a diagnosis of high-grade intraepithelial neoplasia (**Figures 1C, D**). Within a short interval, however, endoscopic recurrence became evident, and repeat resection in September 2024 revealed well-differentiated intramucosal adenocarcinoma. Despite negative deep margins and the absence of lymphovascular or perineural invasion, colonoscopy one month later identified a new 1.5 cm elevated lesion at the same site (**Figures 1E, F**). The tempo of recurrence appeared discordant with the pathological findings obtained from the preceding resections and raised concern that the biological behavior of the lesion had been underestimated.

**Figure 2:**
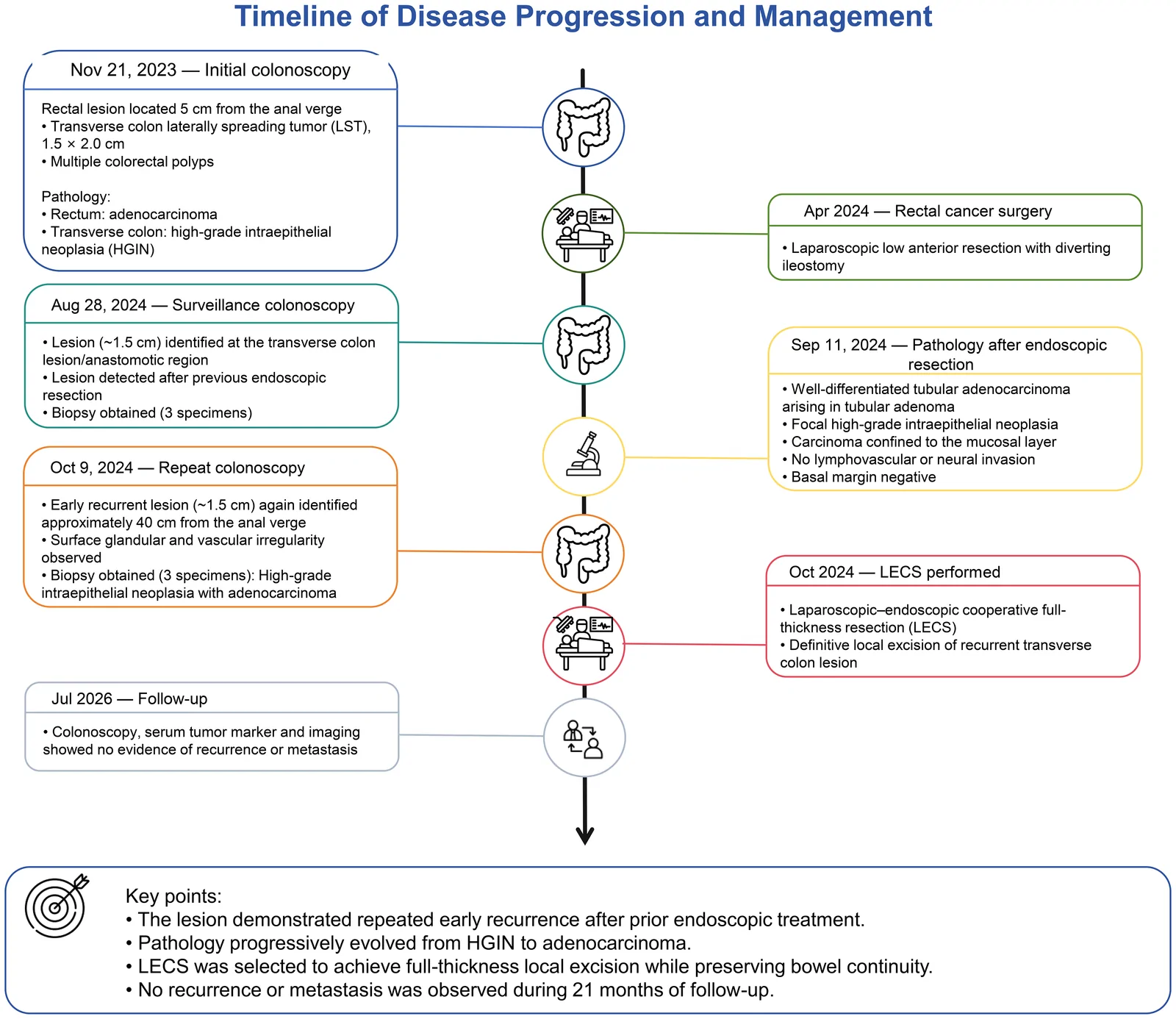
Timeline of clinical course, endoscopic findings, pathological progression, and interventions.

Reassessment at our center demonstrated a 2.0 cm irregularly elevated lesion located about 40 cm from the anal verge (**Figures 1G, H**). Serum carcinoembryonic antigen and carbohydrate antigen 19–9 levels were within normal limits, while contrast-enhanced computed tomography showed neither regional lymphadenopathy nor distant metastatic disease (**Figure 2** and **Supplementary Video 2**). Given the repeated local recurrence, the absence of radiographic evidence of advanced disease, and the previously reported superficial pathological findings, the multidisciplinary team elected to pursue laparoscopic-endoscopic full-thickness resection as both a diagnostic and therapeutic procedure.

**Figure 3:**
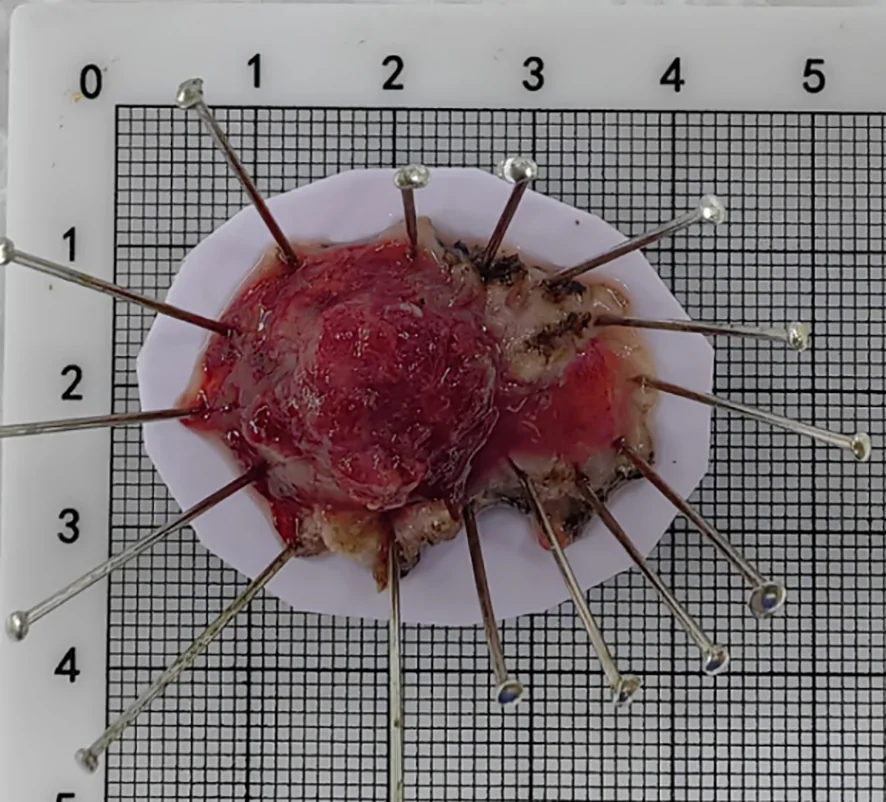
Gross specimen of the resected transverse colon lesion.

The procedure was performed on October 28, 2024, under general anesthesia with the patient in the lithotomy position. A five-port laparoscopic approach was used, and pneumoperitoneum was maintained at 12 mmHg. Laparoscopic exploration revealed a small amount of clear serous ascites but no visible peritoneal, hepatic, or other intra-abdominal metastases. After adhesiolysis and mobilization of the transverse colon, intraoperative colonoscopy identified a 2.0-cm elevated lesion about 40 cm from the anal verge. Narrow-band imaging showed irregular glandular and vascular patterns. A retained clip distal to the lesion was removed, and the lesion was localized using endoscopic transillumination and laparoscopic external compression. Under laparoscopic guidance, an endoscopic hook knife was used to create a circumferential full-thickness incision approximately 5 mm from the lesion. The lesion was then resected en bloc, and the specimen was immediately placed in a protective retrieval bag. The colonic defect was closed laparoscopically with a continuous 3–0 barbed suture and additional seromuscular reinforcement. Repeat colonoscopy confirmed hemostasis, luminal patency, and the absence of narrowing. Estimated blood loss was approximately 20 ml. (**Video 1** and **Figure 3**). The operation was performed on October 28, 2024. The postoperative course was uneventful, and the patient had passed flatus and stool and was tolerating a liquid diet at discharge. The patient was transferred to a community hospital for continued rehabilitation on November 1, 2024, corresponding to postoperative day 4.

Definitive histopathological examination showed moderately differentiated adenocarcinoma extending into the subserosa, corresponding to pT3 disease. The horizontal and deep margins were negative. No lymphovascular or perineural invasion was identified, tumor budding was low grade, and mismatch repair protein expression remained intact. Because no regional lymph nodes were removed, nodal staging was unavailable.

The unexpected pT3 diagnosis prompted repeat multidisciplinary review by colorectal surgery, medical oncology, gastroenterology, radiology, and pathology. The team agreed that completion oncologic colectomy with regional lymphadenectomy remained the standard treatment because local full-thickness resection did not provide nodal staging or address possible occult nodal disease. Negative margins, low-grade tumor budding, absence of lymphovascular and perineural invasion, normal tumor-marker levels, and no radiographic evidence of residual, nodal, or distant disease were considered favorable. However, these indings could not exclude occult nodal metastasis. The potential oncological benefit of further surgery was therefore weighed against the expected functional burden in a patient with a previous ultra-low colorectal anastomosis, including impaired bowel function and possible permanent stoma formation.

After discussion of the standard recommendation, residual nodal risk, and the risks and benefits of further surgery, the patient preferred to preserve bowel continuity and avoid additional resection. Intensive surveillance was therefore chosen as an exceptional, individualized strategy rather than an alternative to standard treatment for pT3 colon cancer.

Surveillance included serial imaging, colonoscopy, and tumor marker monitoring. Ileostomy closure was performed in April 2025. At the latest follow-up in July 2026, approximately 21 months after resection, no local or distant recurrence had been detected (**Supplementary Figure 1–2** and **Supplementary Videos 3-5**). This remains an interim finding and does not establish long-term oncological control. The patient remains under close surveillance. An incisional hernia later developed at the ileostomy-closure site, and elective repair is planned.

## Discussion

The main contribution of this case lies not in the use of laparoscopic-endoscopic cooperative full-thickness resection (LECS) itself, which has already been described for selected complex colorectal lesions, but in the unusual clinicopathological sequence (1, 8, 9). Repeated endoscopic specimens showed only high-grade intraepithelial neoplasia or intramucosal adenocarcinoma, whereas definitive full-thickness resection unexpectedly revealed subserosal invasion, corresponding to pT3Nx disease. This discordance created a second clinical dilemma: whether completion oncologic colectomy with regional lymphadenectomy should be performed in a patient with a previous ultra-low colorectal anastomosis and substantial concerns regarding the functional consequences of further colorectal surgery (10, 11).

Most previous reports of colorectal LECS have focused on technical feasibility, preservation of bowel length, avoidance of unnecessary segmental colectomy, and short-term perioperative outcomes in lesions unsuitable for conventional endoscopic removal. By contrast, the present case highlights the diagnostic significance of rapid recurrence despite apparently favorable superficial pathology and the management uncertainty that arises when definitive local excision reveals unexpectedly advanced invasive disease. The subsequent decision not to perform completion colectomy was exceptional and patient-specific. It should not be interpreted as an extension of the usual indications for colorectal LECS or as an alternative standard for pT3 colon cancer.

The clinical course observed in the present patient highlights this concern. Successive pathological evaluations classified the lesion as high-grade intraepithelial neoplasia and subsequently intramucosal adenocarcinoma, findings that would ordinarily predict a low likelihood of aggressive behavior. The pattern of recurrence, however, appeared disproportionate to these pathological assessments. Despite the absence of radiological evidence of advanced disease, repeated regrowth within a short interval raised suspicion that the lesion had been systematically underestimated (12, 13). Definitive full-thickness excision ultimately demonstrated subserosal invasion, revealing a degree of pathological discordance that was not apparent from either imaging or prior endoscopic specimens. Although causal inferences cannot be drawn from a single observation, the case suggests that rapidly recurrent lesions may occasionally harbor more advanced disease than initial sampling indicates.

Recent systematic evidence supports hybrid laparoscopic-endoscopic approaches as bowel-preserving options for complex colorectal lesions that cannot be removed safely or completely by conventional endoscopic techniques. Distefano et al. reviewed 27 studies involving 1, 112 patients treated with heterogeneous hybrid procedures, including laparoscopically assisted endoscopic resection and endoscopy-assisted laparoscopic wedge or full-thickness resection. The pooled estimates were 5% for additional surgery, 12% for adenocarcinoma identified in the resected specimen, 7% for subsequent surgery performed for oncological indications, and 3% for local recurrence, although different studies contributed to each outcome (14). These findings support the technical feasibility of hybrid resection and suggest that invasive malignancy may remain unrecognized before resection in a clinically relevant proportion of complex lesions.

Nevertheless, these results should be applied cautiously to the present patient. The review combined different procedures, lesion types, selection criteria, and indications for further surgery. Follow-up was reported in only 18 of the 27 studies, with a median duration of 20 months, and long-term disease-free and overall survival were inconsistently documented. Current evidence therefore supports colorectal LECS mainly for carefully selected lesions without definite evidence of deeply invasive or node-positive disease. It does not establish oncological equivalence between local hybrid resection and formal colectomy, particularly when final pathology demonstrates pT3 disease and regional lymph nodes have not been assessed.

Representative recent studies are summarized in **Table 1** (10, 15–23). Despite differences in terminology and technique, the principal indications were broadly similar: large or anatomically difficult lesions, non-lifting or fibrotic lesions, residual or recurrent neoplasia after previous endoscopic treatment, periappendiceal or cecal lesions, and lesions requiring en bloc or full-thickness excision for reliable histopathological assessment. Short-term technical and perioperative outcomes were generally favorable. However, most studies were retrospective, involved highly selected patients treated at experienced centers, and used heterogeneous outcome definitions and operative methods. The main oncological limitation is that local hybrid resection does not provide regional lymph-node staging or clearance. Exposure-type full-thickness resection may also permit luminal contamination or direct tumor exposure to the peritoneal cavity. Unexpected invasive carcinoma on final pathology may therefore require completion oncologic resection.

**Table 1:** Summary of published LECS-related approaches for complex colorectal lesions.

Author, year	Patients, n	Lesion characteristics	LECS-related technique	Malignancy identified	Management after pathology	Follow-up oncologic outcome
Huang et al. (15)	47 overall; 9 treated with endoscopic-laparoscopic approach	Periappendiceal, cecal, or appendiceal adenomas involving the appendiceal orifice	EMR, extended laparoscopic appendectomy, or combined EMR plus extended laparoscopic appendectomy	No invasive cancer reported; mainly adenomatous/serrated	No patient required ileocecal resection	Recurrence/metastasis not reported
Golda et al. (10)	46 overall; 23 treated with CELS	Endoscopically unresectable presumed benign colonic polyps	CELS, mainly endoscopic-assisted laparoscopic local resection	Invasive adenocarcinoma 4/46 overall; 3 after CELS	Patients with invasive adenocarcinoma after CELS were advised to undergo oncologic resection	Recurrence/metastasis not reported; no postoperative mortality.
Jones et al. (16)	37	Complex colonic polyps unsuitable for conventional endoscopic resection	Combined endoscopic robotic surgery	Signet-ring cell adenocarcinoma 1/37; dysplasia 2/37	Not specified	Recurrence/metastasis not reported; 30-day outcomes only.
Parker et al. (17)	55	Complex colonic polyps unsuitable for endoscopic treatment alone after MDT review	Laparoscopically assisted EMR	Cancer 6/55	Three procedures were converted to bowel resection for suspected malignancy; three cancers diagnosed on final histology underwent later laparoscopic bowel resection	Median follow-up 76 months; benign residual/recurrent disease in 7/44 patients without bowel resection, all treated endoscopically; metastasis not reported.
Serra-Aracil et al. (18)	17	Complex benign colonic polyps, most commonly located in the cecum	CELS, including EAWR, LEAR/LEAP, and ECSR	Malignancy 4/17	Oncologic colectomy was performed when local excision was not feasible or pT2 disease was found; one high-risk patient did not undergo second oncologic surgery because of comorbidity	Follow-up colonoscopy was planned; recurrence/metastasis not reported.
Kolosov et al. (19)	51 overall; 31 hybrid laparo-endoscopic surgery	Endoscopically irremovable colon adenomas/tumors	Hybrid laparo-endoscopic surgery	Invasive T1 cancer 5/31 in hybrid group	Not specified in the extracted text	Recurrence/metastasis not reported; early postoperative outcomes only.
Leicher et al. (20)	118 overall; 110 successful CAL-WR	Endoscopically inaccessible, non-lifting/recurrent lesions, or uncertain pT1 margins	Modified colonoscopic-assisted laparoscopic wedge resection	Malignant histology 22/110 successful CAL-WR, including pT1-pT3 cancers	Additional oncologic segmental resection was performed in 12 cases (11%)	Residual scar tissue was observed in 5% during endoscopic follow-up; metastasis not reported.
Hanevelt et al. (21)	57	Suspected high-grade dysplasia or T1 colon cancer requiring en bloc full-thickness local resection	Colonoscopy-assisted laparoscopic wedge resection	Benign 19/57; pT1 cancer 27/57; pT2 cancer 11/57	Completion oncologic resection was advised in 17 patients; 11 underwent completion surgery and 6 chose close surveillance after shared decision-making, mainly because of comorbidity	Follow-up data were available for 17/27 pT1 cases; no recurrence was detected on follow-up colonoscopy; lymph-node metastasis was found in 1 pT2 case after completion surgery.
Bulut et al. (22)	97	Complex colonic lesions selected by benign or malignant MDT; lesions predominantly right-sided	MDT-guided CELS using EA-WR or LA-EMR, with step-up segmental resection when required	Adenocarcinoma 15/97	Treatment was individualized according to intraoperative suspicion and final pathology, including step-up segmental resection or surveillance in selected cases	Benign recurrence rate 4%; distant metastasis not reported.

CAL-WR, colonoscopy-assisted laparoscopic wedge resection; CELS, combined endoscopic laparoscopic surgery; EA-WR, endoscopic-assisted wedge resection; ECSR, endoscopic-assisted segmental resection; EMR, endoscopic mucosal resection; FLEX, full-thickness laparoendoscopic excision; LA-EMR, laparoscopic-assisted endoscopic mucosal resection; LEAR/LEAP, laparoscopic endoscopic-assisted resection/polypectomy; MDT, multidisciplinary team.

This course suggests several practical clinical lessons. Repeated early regrowth at the same site after apparently complete endoscopic resection should not automatically be attributed to residual superficial neoplasia, especially when previous specimens are fragmented, superficial, or affected by treatment-related artifact. A clear mismatch between the tempo of recurrence and the reported pathology should prompt re-review of earlier specimens, multidisciplinary reassessment, and reconsideration of whether the deepest component of the lesion has been adequately evaluated. When concern for deep mural invasion persists, obtaining a definitive en bloc specimen may be appropriate. If nodal-risk cancer is suspected, however, standard oncologic resection remains the preferred treatment rather than repeated endoscopic retreatment. In the present patient, LECS was selected because previous specimens suggested mucosa-confined disease and imaging showed no macroscopic nodal or distant spread, yet the recurrent behavior and endoscopic appearance were difficult to reconcile with superficial neoplasia alone (24, 25). Full-thickness resection therefore offered both local treatment and definitive pathological assessment while preserving bowel continuity (13, 26). The procedure achieved local R0 excision and established the true depth of invasion (11, 27). However, because no regional lymph nodes were removed, the final pathological classification was pT3Nx. The technical success of the procedure therefore did not establish oncological completeness.

Current guideline-based management recommends oncologic colectomy with lymphadenectomy for pT3 colon cancer (3, 28). Local full-thickness resection neither stages nor treats occult nodal disease. Completion colectomy was therefore presented to the patient as the standard oncological option. The multidisciplinary discussion also considered the negative margins, low-grade tumor budding, absence of lymphovascular and perineural invasion, normal tumor-marker levels, and lack of radiographic nodal or distant disease (29). These findings were favorable but did not exclude occult nodal metastasis. The patient had previously received XELOX for rectal cancer, although its effect on the transverse-colon lesion and any occult nodal disease remained uncertain.

Functional considerations were also important. The patient had undergone low anterior resection with an ultra-low colorectal anastomosis. The multidisciplinary team anticipated that further colonic resection and another anastomosis could increase operative morbidity, worsen bowel function, and increase the possibility of permanent diversion (12, 28, 30). After being informed that completion colectomy remained the standard treatment and that surveillance carried an unresolved risk of occult nodal disease, the patient declined further surgery. Intensive surveillance was adopted as an exceptional, individualized strategy rather than as an alternative standard of care.

No local or distant recurrence has been detected during approximately 21 months of follow-up. This interval, however, is insufficient to establish durable oncological control, particularly because pathological nodal status remains unknown. The current outcome should therefore be regarded as an interim observation. Continued close surveillance is necessary, and whether rapid clinicopathological discordance can prospectively identify lesions requiring earlier oncologic resection remains unresolved.

## Patient perspective

The patient identified preservation of bowel continuity and avoidance of permanent stoma formation as major treatment priorities. He understood that completion oncologic colectomy with regional lymphadenectomy remained the standard treatment and that surveillance alone carried an unresolved risk of occult nodal disease and subsequent recurrence. After discussing these uncertainties and the potential functional consequences of additional colorectal resection, he elected to proceed with intensive surveillance. At the most recent follow-up in July 2026, the patient reported satisfactory functional recovery without substantial limitations in daily activities. He understood that the absence of recurrence at approximately 21 months represented an interim finding and that prolonged surveillance remained necessary because the long-term oncological outcome was uncertain.

## Conclusion

This case highlights the potential discordance between repeated endoscopic pathology and the underlying biological behavior of a colorectal lesion. Despite serial pathological findings of high-grade intraepithelial neoplasia and intramucosal adenocarcinoma, rapid recurrence ultimately revealed pT3 disease after full-thickness excision. The clinical course raises the possibility that recurrent behavior may warrant additional diagnostic scrutiny even when pathological and radiological findings appear reassuring.

Management in this patient incorporated pathological findings, prior treatment history, anticipated functional outcomes, and patient preference rather than pathological stage alone. Laparoscopic-endoscopic cooperative full-thickness resection achieved complete local excision and definitive pathological assessment while preserving bowel continuity.

The absence of recurrence during 21 months of follow-up should be interpreted cautiously. Regional lymphadenectomy was not performed, occult nodal disease cannot be excluded, and the duration of surveillance remains insufficient to establish long-term oncological adequacy. This exceptional management decision should therefore not be interpreted as supporting laparoscopic-endoscopic cooperative full-thickness resection as an alternative to standard oncologic colectomy with regional lymphadenectomy for pT3 colon cancer.

## Data Availability

The original contributions presented in the study are included in the article/**Supplementary Material**. Further inquiries can be directed to the corresponding authors.
