# Supplementary material for: Case Report: Laparoscopic-endoscopic cooperative full-thickness resection for recurrent transverse colon cancer after rectal cancer surgery: a case report with video
Source: Front Oncol. 2026 Aug 7;16:1908320. doi: 10.3389/fonc.2026.1908320 (PMC13494701; doi:10.3389/fonc.2026.1908320)
Supplement: Supplementary file 1 [file SupplementaryFile1.docx]

**Supplementary Video 1.** Preoperative contrast-enhanced abdominal computed tomography before rectal cancer surgery.

**Supplementary Video 2.** Preoperative contrast-enhanced abdominal computed tomography before transverse colon surgery.

**Supplementary Video 3.** Postoperative contrast-enhanced abdominal computed tomography in October 2024.

**Supplementary Video 4.** Postoperative contrast-enhanced abdominal computed tomography in November 2025.

**Supplementary Video 5.** Follow-up abdominal computed tomography performed in July 2026 showing no evidence of disease recurrence.

A

B

**Supplementary Figure 1. （A）Carcinoembryonic antigen levels at three time points: before transverse colon surgery, April 2025, and November 2025; （B）Carbohydrate antigen 19-9 levels at the same time points.**

**Supplementary Figure 2.** Follow-up colonoscopic findings after full-thickness resection. (A) Colonoscopic image obtained in February 2025. (B) Colonoscopic image obtained in November 2025.
